# Acceleration of cellodextrin phosphorolysis for bioelectricity generation from cellulosic biomass by integrating a synthetic two-enzyme complex into an in vitro synthetic enzymatic biosystem

**DOI:** 10.1186/s13068-019-1607-4

**Published:** 2019-11-12

**Authors:** Dongdong Meng, Ranran Wu, Juan Wang, Zhiguang Zhu, Chun You

**Affiliations:** 1 Tianjin Institute of Industrial Biotechnology, Chinese Academy of Sciences, 32 West 7th Avenue, Tianjin Airport Economic Area, Tianjin, 300308 People’s Republic of China; 2 University of Chinese Academy of Sciences, 19A Yuquan Road, Shijingshan District, Beijing 100049 People’s Republic of China

**Keywords:** Cascade enzymes, Synthetic enzyme complex, Cellodextrin, In vitro synthetic enzymatic biosystem, Bioelectricity

## Abstract

**Background:**

Cellulosic biomass, the earth’s most abundant renewable resource, can be used as substrates for biomanufacturing biofuels or biochemicals via in vitro synthetic enzymatic biosystems in which the first step is the enzymatic phosphorolysis of cellodextrin to glucose 1-phosphate (G1P) by cellodextrin phosphorylase (CDP). However, almost all the CDPs prefer cellodextrin synthesis to phosphorolysis, resulting in the low reaction rate of cellodextrin phosphorolysis for biomanufacturing.

**Results:**

To increase the reaction rate of cellodextrin phosphorolysis, synthetic enzyme complexes containing CDP and phosphoglucomutase (PGM) were constructed to convert G1P to glucose 6-phosphate (G6P) rapidly, which is an important intermediate for biomanufacturing. Four self-assembled synthetic enzyme complexes were constructed with different spatial organizations based on the high-affinity and high-specific interaction between cohesins and dockerins from natural cellulosomes. Thus, the CDP–PGM enzyme complex with the highest enhancement of initial reaction rate was integrated into an in vitro synthetic enzymatic biosystem for generating bioelectricity from cellodextrin. The in vitro biosystem containing the best CDP–PGM enzyme complex exhibited a much higher current density (3.35-fold) and power density (2.14-fold) than its counterpart biosystem containing free CDP and PGM mixture.

**Conclusions:**

Hereby, we first reported bioelectricity generation from cellulosic biomass via in vitro synthetic enzymatic biosystems. This work provided a strategy of how to link non-energetically favorable reaction (cellodextrin phosphorolysis) and energetically favorable reaction (G1P to G6P) together to circumvent unfavorable reaction equilibrium and shed light on improving the reaction efficiency of in vitro synthetic enzymatic biosystems through the construction of synthetic enzyme complexes.

## Background

Cellulose is the most abundant and renewable bioresource on earth, and it has long been considered to be a potential sustainable source sufficient for the production of biofuels and biochemicals [1]. Cellulose is a linear polymer of anhydroglucose units linked by beta-1,4-glycosidic bonds and accounts for 35–50% of the lignocellulosic biomass in dry weight [2]. Cellulosic biomass utilization usually includes biomass pretreatment to cellodextrin or regenerated cellulose, enzymatic hydrolysis to fermentative glucose catalyzed by a mixture of cellulases [including endoglucanase (EC 3.2.1.4), cellobiohydrolase (EC 3.2.1.91), β-glucosidase (EC 3.2.1.21)] and sugar fermentation by microorganisms to products [3, 4]. Cellodextrins, which are water-soluble oligosaccharides with degrees of polymerization from two to six, can be prepared by the incomplete acid hydrolysis of cellulose [5] or enzymatic synthesis from glucose 1-phosphate (G1P) by cellodextrin phosphorylase (CDP, EC 2.4.1.49) [6]. CDP can also catalyze the phosphorolysis of cellodextrins to G1P. This family 94 glycoside hydrolase (GH) exhibits a very important function in intracellular cellodextrin metabolism in anaerobic cellulolytic bacteria such as *Clostridium thermocellum* [7]. Cellodextrins and CDP can be designed in many in vitro synthetic enzymatic biosystems by replacing starch and starch phosphorylase to produce hydrogen [8], bioelectricity [9] and value added chemicals [10].

The in vitro synthetic enzymatic biosystem is a new emerging powerful platform that can be prepared by mixing a number of enzymes and cofactors for biomanufacturing purposes without cellular constraints [11, 12]. Compared to in vivo whole-cell fermentation, which is the predominant biomanufacturing platform, in vitro synthetic enzymatic biosystems have numerous distinctive advantages [12, 13], such as high product yield [14], fast reaction rate in enzymatic fuel cells ascribed to unobstructed mass transfer [15], high engineering flexibility [16] and high tolerance in toxic environments [17]. In vitro synthetic enzymatic biosystems have been used for the synthesis of special proteins and polysaccharides, as well as the economical production of biofuels, biochemicals and potential food/feed from biomass sugars [10–13, 17, 18]. Recently, an in vitro synthetic enzymatic biosystem containing CDP was constructed to covert cellodextrins to inositol in 98% (w/w) yield [19]. Although nearly all CDPs characterized prefer cellodextrin synthesis to phosphorolysis [20], the excellent downstream exergonic reactions (the Gibbs free energies of converting G1P to glucose 6-phosphate (G6P), converting G6P to inositol 1-phosphate (I1P) and converting I1P to inositol are − 7.4 ± 1.5, − 50.3 ± 9.7 and − 20.7 ± 9.8 kJ mol^−1^, respectively) in inositol production pathway push the overall reaction toward completeness. However, most of the downstream reactions in other in vitro synthetic enzymatic biosystems do not have these kinds of negative Gibbs free energy values, such as hydrogen and bioelectricity production pathway (the Gibbs free energy of converting G6P to 6-phospho-d-glucono-1,5-lactone (6PG) is only − 2.3 kJ mol^−1^) [19], resulting in the inefficient utilization of cellodextrins. In addition, CDP exhibited fivefold higher *k*_cat_ value and twofold higher catalytic efficiency (*k*_cat_/*K*_m_) value against cellotetraose on synthesis direction than those on phosphorolysis direction [21], indicating the limited activity for cellodextrins utilization of CDPs. Collectively, the non-energetically favorable reaction and non-effective phosphorolysis activity of CDPs may lead to a difficult situation of conversion of cellodextrins to bioelectricity.

In nature, many enzymes are often assembled into enzyme complexes to circumvent unfavorable reaction equilibrium and enhance the reaction rate [22]. Such enzyme complexes could enhance reaction rate due to the process of transferring the product of one enzyme to an adjacent cascade enzyme without full equilibration with the bulk phase [22–25]. Inspired by natural multi-enzyme complexes, constructing synthetic enzyme complexes containing multiple cascade enzymes as building modules can be regarded as a powerful tool in synthetic biology systems, regardless of whether they occur in vivo or in vitro [26–30]. To the in vitro synthetic enzymatic biosystems for bioelectricity production, the strategy of constructing enzyme complexes may be crucial because the power and current density (reaction rate) of the biosystem are usually limited by the diffusion of substrate/intermediates among enzymes. For example, a Krebs cycle metabolon-catalyzed pyruvate/air biofuel cells was constructed by the assembly of various natural enzymes in one matabolon to improve its current and power density [31]. A three-dimensional carbon nanotube combined with DNA scaffold or protein aggregates was also successfully applied for enzyme complex assembly in enzymatic biofuel cells [32–34]. A physically cross-linked enzyme hydrogel containing three dehydrogenases was constructed to achieve the complete oxidation of methanol in biofuel cells [35]. Thus, integrating enzyme complexes containing CDP and its downstream enzyme into the in vitro synthetic enzymatic biosystem for bioelectricity generation maybe a promising strategy to drive the cellodextrin phosphorolysis and as a result to accelerate the bioelectricity generation rate of multienzyme-mediated biosystems.

In this study, different spatial organizations of two-enzyme complexes containing CDP and phosphoglucomutase (PGM, EC 5.4.2.2), which can convert G1P to G6P, were constructed through the species-specific interaction between dockerins and cohesins from natural cellulosomes. The enhancements of initial reaction rates of these two-enzyme complexes were determined. The best enzyme complex yielding the highest enhancement in initial reaction rate was integrated into an in vitro synthetic enzymatic biosystem for generating bioelectricity from cellodextrin, showing much higher current density and power density than that via non-complexed enzyme mixture. The strategy of constructing of a synthetic enzyme complex containing rate-limited enzymes may become a general tool to increase the efficiency of in vitro synthetic enzymatic biosystems.

## Results

### Designing strategy to accelerate cellulose phosphorolysis

**Fig. 1:**
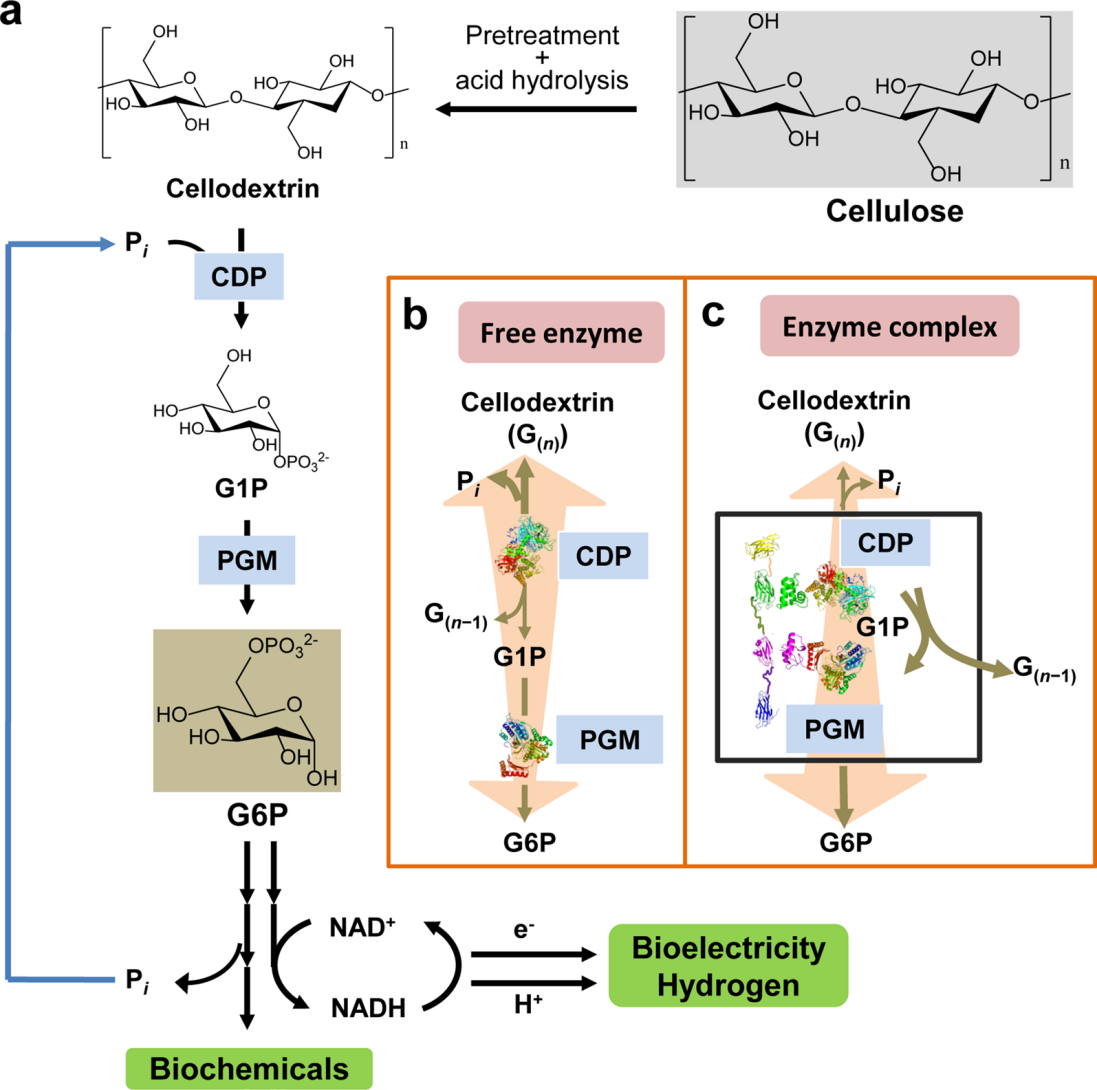
Reaction scheme of cellulose phosphorolysis. **a** Cellulosic biomass was enzymatic phosphorylated via in vitro synthetic enzymatic biosystems to manufacturing biochemicals or biofuels. **b** In the free enzyme mixture, CDP prefers to convert cellodextrin with a degree of polymerization of *n* − 1 (G_(*n*−1)_) and G1P to G_(*n*)_ and inorganic phosphate (P_*i*_) despite the presence of PGM. **c** The enzyme complex containing CDP and PGM was supposed to accelerate the degradation of cellodextrin to G6P. *CDP* cellodextrin phosphorylase, *PGM* phosphoglucomutase, *G*_*(n)*_ *and G*_*(n−1)*_ cellodextrin, *G1P* glucose 1-phosphate, *G6P* glucose 6-phosphate, *P*_*i*_ inorganic phosphate

In the in vitro synthetic enzymatic biosystems powered by cellodextrin (Fig. 1a), CDP is responsible for the phosphorolysis of cellodextrin with the degree of polymerization (DP) of *n* (G_(*n*)_) to G1P and G_(*n*−1)_ in the presence of phosphate. Cellodextrin can be degraded by CDP continually until the remaining cellodextrin residues are cellobiose [19]. Then PGM converts G1P to G6P, which is an important intermediate for many in vitro synthetic enzymatic biosystems to manufacture biochemicals and biofuels. According to thermodynamic analysis of the reactions catalyzed by CDP and PGM [36], Gibbs free energies of converting cellohexaose and phosphate to cellopentaose and G1P by CDP and converting G1P to G6P by PGM are 3.2 ± 3.6 and − 7.4 ± 1.5 kJ mol^−1^ (Additional file 1: Table S1), respectively. It seems that the glucosyl units in cellodextrin can be converted to G6P efficiently via cascade reactions catalyzed by combination of CDP and PGM. However, the activity of CDP on phosphorolysis direction is much lower than that on synthesis direction [37] and the number of PGM molecules is much fewer than that of CDP under the same unit loading because of the much higher activity of PGM than CDP. Thus, the G1P molecules generated from the phosphorolytic cleavage of cellodextrins may be taken back by CDP itself for cellodextrin synthesis before reaching the PGM molecule to generate G6P (Fig. 1b), resulting in a slow reaction rate for G6P production. On the contrary, inspired by the successful examples of increase of the overall reaction rate and efficiency by enzyme complexes [38], the construction of CDP–PGM enzyme complex can be considered to increase the reaction rate of cellodextrin phosphorolysis, accelerating G6P production to feed downstream enzymes for efficient biomanufacturing (Fig. 1c).

### Construction of synthetic enzyme complexes with different enzyme spatial organizations

**Fig. 2:**
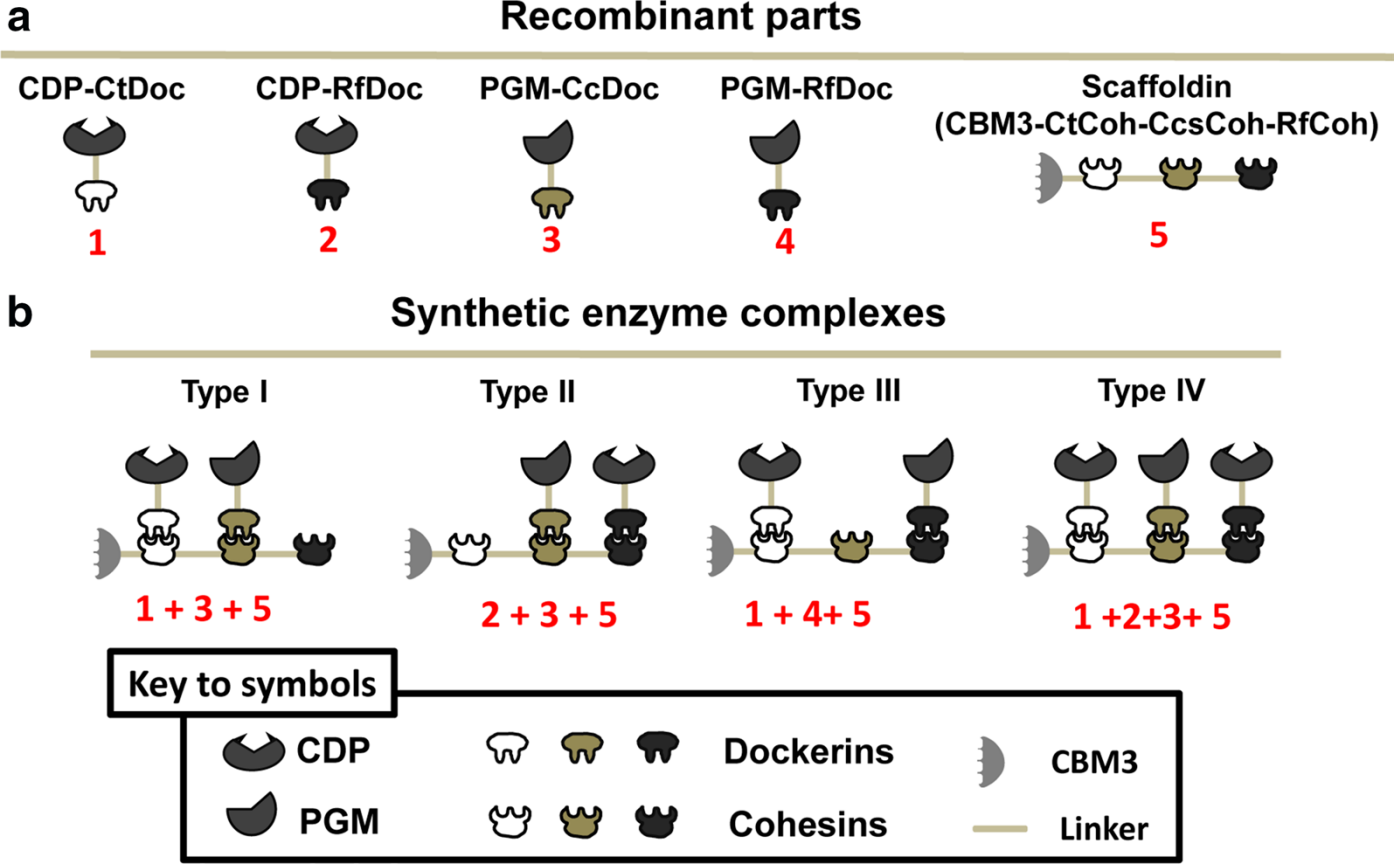
Schematic representation of the chimeric enzymes and four types of enzyme complexes. **a** Schematic representation of dockerin-tagged recombinant proteins and cohesin-containing scaffoldin. Two dockerins containing CDPs (part 1 and 2), two dockerins containing PGM (part 3 and 4), and a synthetic scaffoldin (part 5) were constructed. **b** Types of synthetic enzyme complexes. The type I CDP–PGM complex was constructed by CDP-CtDoc, PGM-CcsDoc and CBM3-Scaf3 (parts 1 + 3 + 5); the type II complex was constructed by CDP-RfDoc, PGM-CcsDoc and CBM3-Scaf3 (parts 2 + 3 + 5); the type III complex was constructed by CDP-CtDoc, PGM-RfDoc and CBM3-Scaf3 (parts 1 + 4 + 5); and the type IV complex was constructed by CDP-CtDoc, CDP-RfDoc, PGM-CcsDoc and CBM3-Scaf3 (parts 1 + 2 + 3 + 5)

To increase the reaction rate of cellodextrin phosphorolysis, several synthetic enzyme complexes with different CDP–PGM organizations in a synthetic mini-scaffoldin were constructed according to Bayer’s proposal [39] about constructing designed enzyme complexes utilizing species-specificity dockerins and cohesins from the natural cellulosomes (Fig. 2a). CDP from *C. thermocellum* was fused with a dockerin from *C. thermocellum* CelS (CtDoc) and *Ruminococcus flavefaciens* ScaA (RfDoc) at its C-terminus to obtain CDP-CtDoc (part 1) and CDP-RfDoc (part 2), respectively. PGM from *Thermococcus kodakaraensis* was fused with a dockerin from *Clostridium cellulovorans* EngE (CcsDoc) and RfDoc at its C-terminus to obtain PGM-CcsDoc (part 3) and PGM-RfDoc (part 4), respectively. The synthetic mini-scaffoldin CBM3-CtCoh-CcsCoh-RfCoh (i.e., CBM3-Scaf3, part 5) was constructed to contain a family 3 cellulose-binding module (CBM3) at the N-terminus followed by three different types of cohesins from the *C. thermocellum* ATCC 27405 CipA, *C. cellulovorans* ATCC 35296 CbpA and *R. flavefaciens* ScaB [40]. Four types of CDP–PGM synthetic enzyme complexes were constructed (Fig. 2b). The type I CDP–PGM complex was constructed by CDP-CtDoc, PGM-CcsDoc and CBM3-Scaf3 (parts 1 + 3 + 5), the type II complex was constructed by CDP-RfDoc, PGM-CcsDoc and CBM3-Scaf3 (parts 2 + 3 + 5), the type III complex was constructed by CDP-CtDoc, PGM-RfDoc and CBM3-Scaf3 (parts 1 + 4 + 5), and the type IV complex was constructed by CDP-CtDoc, CDP-RfDoc, PGM-CcsDoc and CBM3-Scaf3 (parts 1 + 2 + 3 + 5). Such synthetic enzyme complexes can be adsorbed on the surface of cellulosic material through CBM3 in the scaffoldin. After washing and centrifugation, the synthetic enzyme complexes were purified and immobilized on solid regenerated amorphous cellulose (RAC) [40].

**Fig. 3:**
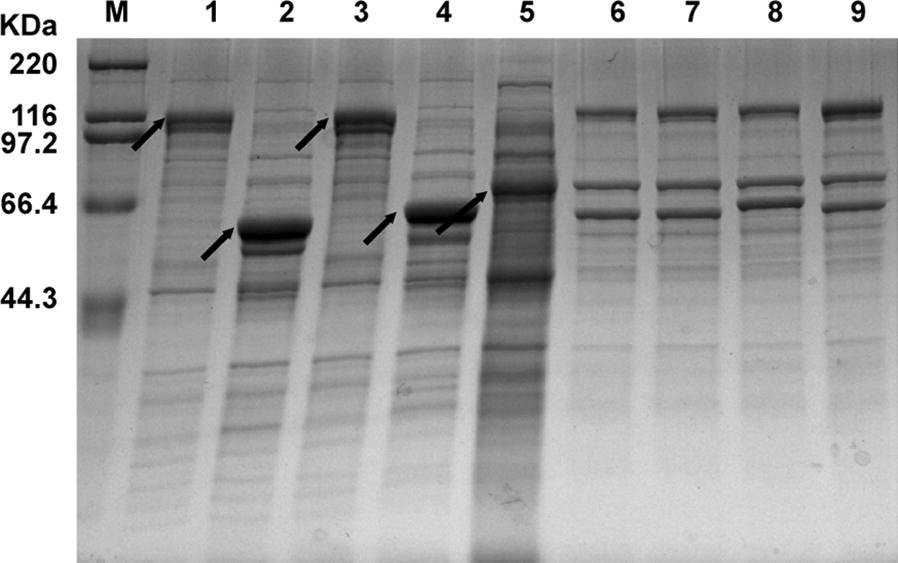
SDS-PAGE analysis of the *E. coli* cell extracts containing the recombinant proteins and synthetic enzyme complexes purified by RAC pull-down. Lanes 1–5, cell extracts containing CDP-CtDoc, PGM-CcsDoc, CDP-RfDoc, PGM-RfDoc and CBM3-Scaf3, respectively; lane 6, RAC absorbed CBM3-Scaf3, CDP-CtDoc and PGM-CcsDoc; lane 7, RAC absorbed CBM3-Scaf3, CDP-RfDoc and PGM-CcsDoc; lane 8, RAC absorbed CBM3-Scaf3, CDP-CtDoc and PGM-RfDoc; lane 9, RAC absorbed CBM3-Scaf3, CDP-CtDoc, PGM-CcsDoc, and CDP-RfDoc

SDS-PAGE analysis was performed to investigate protein expression, purification and co-immobilization of synthetic enzyme complexes (Fig. 3). The five cell lysates containing CDP-CtDoc, PGM-CcsDoc, CDP-RfDoc, PGM-RfDoc and CBM3-Scaf3 are shown in lanes 1–5 of Fig. 3, respectively, and the target bands are indicated by arrows. Synthetic enzyme complexes were constructed by mixing cell lysate supernatant of CBM-Scaf3, cell lysate supernatant of dockerin-containing enzymes and RAC, making sure that dockerin-containing enzymes were in slight excess compared to CBM-Scaf3. The purified co-immobilized four types of synthetic enzyme complexes on RAC are shown in lanes 6–9 of Fig. 3. All four types of synthetic enzyme complexes exhibited three distinct bands, representing CDP, PGM and CBM3-Scaf3. In lanes 6, 7 and 8 for type I, II and III synthetic enzyme complexes, respectively, the band intensity of the three bands indicated that the ratio of scaffoldin, CDP and PGM was approximately 1:1:1. In lane 9 for the type IV complex, the intensity of the top band was about twofold than that of the same bands in lane 6, 7 and 8, indicating that one scaffoldin can bind to two different types of CDP and one PGM in the type IV complex at the same time. The bands of SDS-PAGE indicated that all types of synthetic enzyme complexes were successfully constructed and the molar ratios of CDP and PGM in synthetic enzyme complexes were exactly the same as expected.

### Enzyme activity of CDP and PGM in synthetic enzyme complexes

When comparing the reaction rate of cellodextrin phosphorolysis by enzyme complex and free enzyme mixture, the enzyme unit of CDP and PGM in enzyme complex and free enzyme mixture should be equal. However, the purity of one-step purified enzyme complexes as shown in Fig. 3 was not high enough. Thus, it was difficult to determine the enzyme amount of the CDP and PGM in enzyme complex precisely, resulting in incorrect enzyme unit usage. Besides, because CDP and PGM were cascade enzymes, the individual enzyme activity values of CDP and PGM in enzyme complex containing these two enzymes were also difficult to measure experimentally. However, the maximum amounts of CDP or PGM which attached to certain amount of CBM3-Scaf3 should be constant. These constant values can be represented by the enzyme activity values of CDP or PGM when either of these two enzymes was attached to CBM3-Scaf3 individually. As shown in Additional file 1: Figure S1a, 0.5 mL cell lysate supernatant of CBM3-Scaf3 was mixed with different volume of cell lysate supernatant of CDP-CtDoc, followed by excessive RAC immobilization. When 1 mL supernatant of CDP-CtDoc was mixed with 0.5 mL cell lysate supernatant of CBM3-Scaf3, RAC immobilized CBM3-Scaf3/CDP-CtDoc reach its highest specific activity of 0.72 U mL^−1^. Excess addition of CDP-CtDoc would not increase the activity of RAC immobilized CBM3-Scaf3/CDP-CtDoc. Similarly, when 0.5 mL cell lysate supernatant of CBM3-Scaf3 was mixed with 2 mL supernatant of CDP-RfDoc, 2 mL supernatant of PGM-CcsDoc or 2 mL supernatant of PGM-RfDoc individually, RAC immobilized CBM3-Scaf3/CDP-RfDoc, CBM3-Scaf3/PGM-CcsDoc, and CBM3-Scaf3/PGM-RfDoc reach its highest specific activity of 0.71 U mL^−1^, 23 U mL^−1^ and 22.4 U mL^−1^, respectively (Additional file 1: Figure S1b, c and d). And we also determined the specific activity values of free CDP and PGM. The specific activity of CDP was 97.5 ± 1.8 U μmol^−1^ (0.89 ± 0.07 U mg^−1^) on phosphorolysis direction of cellodextrin (DP is 4.4) at 60 °C. The specific activity of PGM was 2963 ± 143 U μmol^−1^ (59 ± 3 U mg^−1^) on G1P at 60 °C. Based on these activity values, we could adjust the CDP and PGM loading in free enzyme mixture and ensure that CDP and PGM have the same enzymatic units in synthetic enzyme complex and free enzyme mixture when comparing the reaction rate of cellodextrin phosphorolysis.

### Initial reaction rates of synthetic enzyme complexes for cellodextrin phosphorolysis

**Fig. 4:**
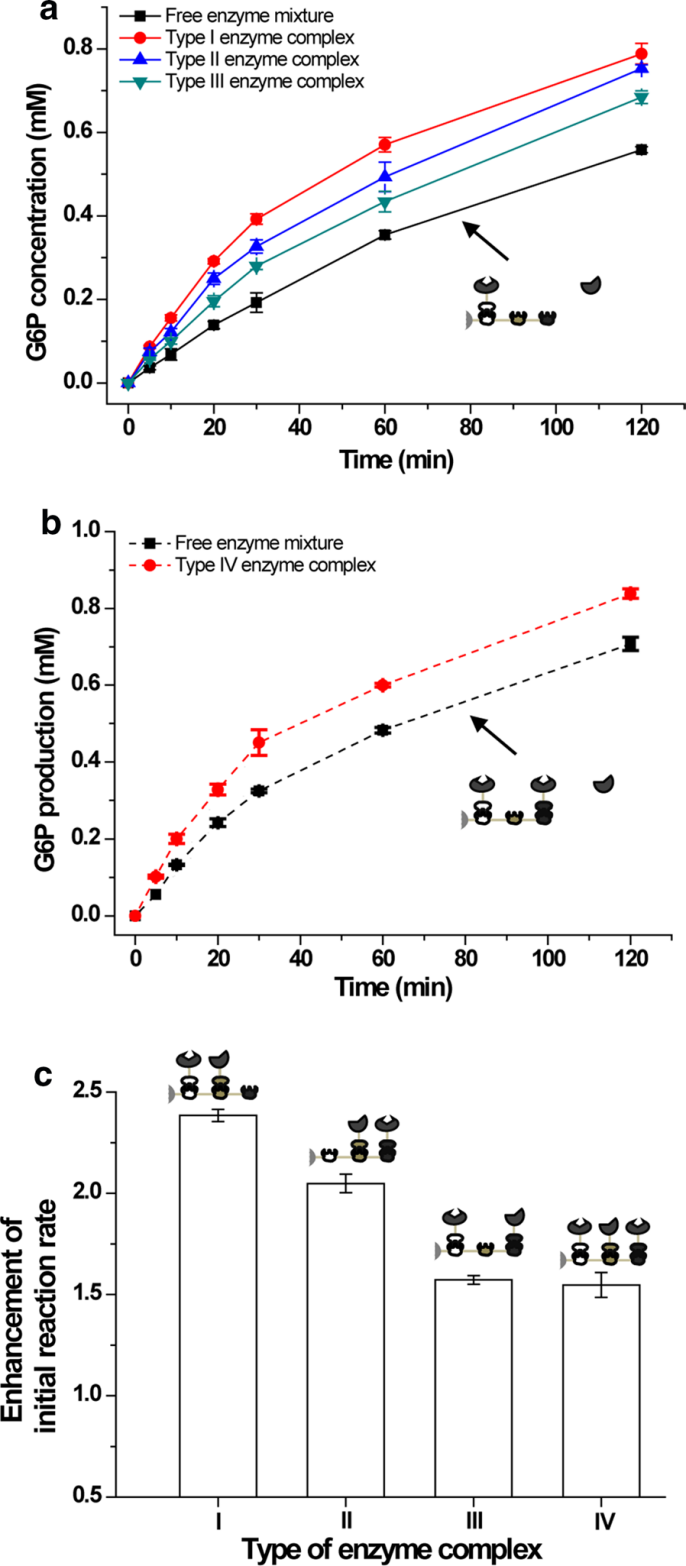
Characterization of different types of synthetic enzyme complexes. **a** Profiles of G6P production catalyzed by type I, II, and III synthetic enzyme complexes and enzyme mixture with the same enzyme unit loading of 8.6 U L^−1^ CDP and 284 U L^−1^ PGM. **b** Profiles of G6P production catalyzed by type IV synthetic enzyme complex and its corresponding enzyme mixture with the same enzyme unit loading of 17.2 U L^−1^ CDP and 284 U L^−1^ PGM. **c** Degree of enhancement in initial reaction rate of four types of synthetic enzyme complexes. Values shown are means of triplicate determinations

The type I enzyme complex immobilized on RAC was eluted by ethylene glycol, and the initial reaction rate of eluted enzyme complex was almost the same with RAC immobilized free enzyme complex, indicating the immobilization of enzymes on the surface of RAC matrix did not significantly affect the enzymatic characteristic of enzyme complexes. Due to the convenient one-step purification and co-immobilization of enzyme complexes, all the RAC immobilized enzyme complexes were used in this study. To diminish the effects of RAC to the reactant, the free enzyme mixture was the mixture of RAC immobilized CBM3-Scaf3/CDP-CtDoc and free his-tagged PGM (Fig. 4a, b).

The initial reaction rates of CDP–PGM synthetic enzyme complex and its corresponding free enzyme mixture were determined at 60 °C on mixed cellodextrin (DP is 4.4) [19] at the same enzyme unit loading, corresponding to 8.6 U L^−1^ CDP and 284 U L^−1^ PGM for type I, II and III synthetic enzyme complex, and 17.2 U L^−1^ CDP and 284 U L^−1^ PGM for type IV synthetic enzyme complex. The initial reaction rate values of G6P produced by the RAC immobilized type I, II and III synthetic enzyme complex were 17.3, 14.9 and 11.4 μM min^−1^, respectively, while the initial reaction rate values of G6P produced by the corresponding free enzyme mixture was 7.3 μM min^−1^ (Fig. 4a). The initial reaction rate of the type IV synthetic enzyme complex was 22.5 μM min^−1^, while the initial reaction rate values of G6P produced by the corresponding free enzyme mixture was 15 μM min^−1^ (Fig. 4b). All the CDP–PGM enzyme complexes showed an increased initial reaction rates. Compared to the corresponding free enzyme mixture, the enhancement values of the initial reaction rate of the type I, II, III and IV synthetic enzyme complex were 2.4, 2.1, 1.6 and 1.5 (Fig. 4c), respectively. The type I synthetic enzyme complex had the most enhanced initial reaction rate among the four types of complexes. The enhancement of the initial reaction rate of the type I synthetic enzyme complex was also determined at a higher enzyme concentration. When the enzyme unit loading was 51.6 U L^−1^ CDP and 1704 U L^−1^ PGM, the type I synthetic complex and its free enzyme mixture exhibited initial reaction rate values of 96.7 and 50.1 μM min^−1^ (Additional file 1: Figure S2a), respectively. The enhancement of initial reaction rate of type I synthetic enzyme complex at 51.6 U L^−1^ CDP and 1704 U L^−1^ PGM enzyme loading is lower than that at 8.6 U L^−1^ CDP and 284 U L^−1^ PGM enzyme loading (Additional file 1: Figure S2b).

The effect of enhancing initial reaction rate via enzyme complexes can be evaluated by adding a competing side reaction into the enzymatic cascade [24]. *Thermotoga maritima* phosphatase (TmPase) catalyzes the dephosphorylation of a wide range of sugar phosphates [41], and its specific activity was determined to be 0.98 U mg^−1^ against G1P and 0.018 U mg^−1^ against G6P under 60 °C, respectively. Therefore, TmPase can be used to add in CDP–PGM cascade to competing consume the intermediate G1P but not consume G6P. In the enzyme mixture and type I enzyme complex biosystem with the enzyme unit loading of 8.6 U L^−1^ CDP and 284 U L^−1^ PGM, different units of TmPase (0 U L^−1^ to 80 U L^−1^) were added and the amounts of G6P were determined after was proceeded for 10 min. The G6P concentration of overall reaction for the enzyme complex biosystem decreased with the increasing of TmPase units, but a weaker extent was observed compared with the enzyme mixture biosystem (Additional file 1: Figure S3), indicating the existence of the proximity effect for enhancing reaction rate via enzyme complexes.

The apparent kinetic parameters of the type I synthetic enzyme complex and its corresponding enzyme mixture were determined at 60 °C based on Michaelis–Menten kinetics. The synthetic enzyme complex exhibited a *K*_m_ value 1.3 times lower and a *k*_cat_ value 2.6 times higher of those of the enzyme mixture against cellodextrin (average DP 4.4) phosphorolysis (Table 1). As a result, the catalytic efficiency (*k*_cat_/*K*_m_) of the enzyme complex was approximately 3.4-fold higher than that of the enzyme mixture, which was consistent with the experimental results of initial reaction rate determination. Considering the cellodextrin used here is a mixture of oligosaccharides, the Michaelis–Menten kinetic parameters against cellopentaose were also determined. Similar to the kinetic parameters against cellodextrin, the *k*_cat_/*K*_m_ value of the type I synthetic enzyme complex was 3.3 times higher than that of the enzyme mixture (Table 1). In addition, *k*_cat_ values of enzyme mixture and type I enzyme complex were determined at other temperatures (45 °C, 50 °C, and 55 °C). Then, the activation energy of enzyme mixture and type I enzyme complex were calculated to be 45.8 kJ mol^−1^ and 32.8 kJ mol^−1^ based on Arrhenius plot (Additional file 1: Table S2), respectively, indicating that the construction of enzyme complexes can increase reaction rate by decreasing activation energy of total enzyme cascade [42].

**Table 1:** Apparent kinetic parameters for the type I synthetic enzyme complex and enzyme mixture at 60 °C

Name	Cellodextrin			Cellopentose		
	*K*_m_ (g L^−1^)	*k*_cat_ (s^−1^)	*k*_cat_/*K*_m_ (s^−1^ g^−1^ L)	*K*_m_ (mM)	*k*_cat_ (s^−1^)	*k*_cat_/*K*_m_ (mM^−1^ s^−1^)
Type I CDP–PGM complex	2.1 ± 0.7	3.9 ± 0.4	1.9	1.9 ± 0.2	4.6 ± 0.2	2.5
Enzyme mixture	2.8 ± 0.3	1.5 ± 0.1	0.6	2.3 ± 0.2	1.7 ± 0.1	0.7

### Enzyme complex enhancing the reaction rate of bioelectricity generation

**Fig. 5:**
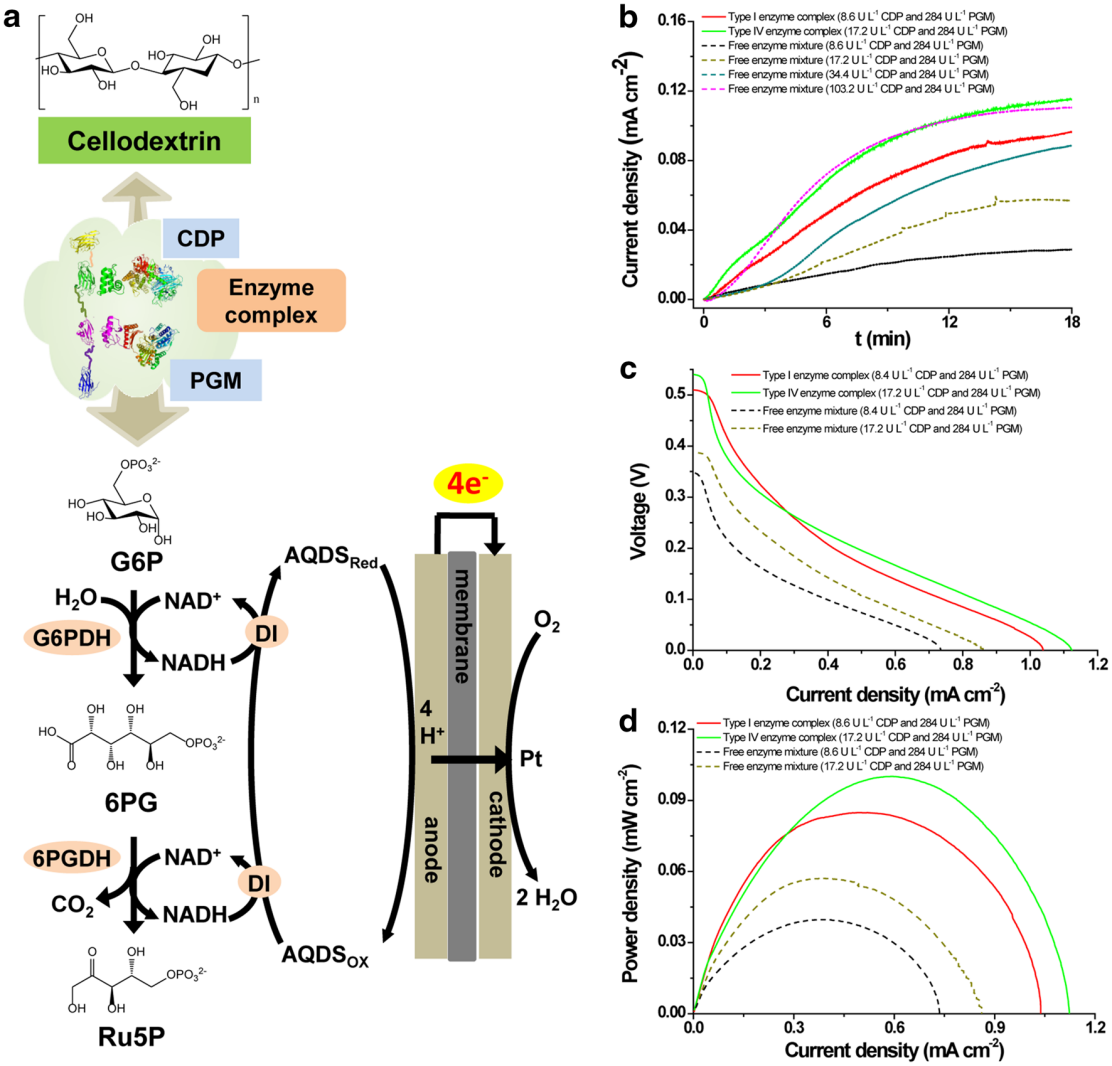
Electrochemical performance of the cellodextrin-powered in vitro synthetic enzymatic biosystem catalyzed by synthetic enzyme complexes and enzyme mixture. **a** Reaction scheme of the in vitro synthetic pathway of the cellodextrin-powered biosystem for bioelectricity generation. *CDP* cellodextrin phosphorylase, *PGM* phosphoglucomutase, *G6PDH* glucose 6-phosphate dehydrogenase, *6PGDH* 6-phosphogluconate dehydrogenase, *DI* diaphorase, *G6P* glucose 6-phosphate, *6PG* 6-phosphogluconate, *Ru5P* ribulose 5-phosphate, *AQDS*_*Red*_ reduced mediator, *AQDS*_*OX*_ oxidized mediator. **b** Amperometric response of the cellodextrin-powered biosystems containing synthetic enzyme complex and enzyme mixture in the initial stage of the reaction. **c** Polarization curves of the cellodextrin-powered biosystems containing synthetic enzyme complex and enzyme mixture. **d** Profiles of power density versus current of the cellodextrin-powered biosystems containing synthetic enzyme complex and enzyme mixture. Type I enzyme complex (red line) was loaded under the enzyme unit of 8.64 U L^−1^ CDP and 284 U L^−1^ PGM. Type IV enzyme complex (green line) was loaded under the enzyme unit of 17.3 U L^−1^ CDP and 284 U L^−1^ PGM. Free enzyme mixture was loaded under the enzyme unit of 8.64 U L^−1^ CDP and 284 U L^−1^ PGM (black line), 17.3 U L^−1^ CDP and 284 U L^−1^ PGM (dark yellow line), 34.6 U L^−1^ CDP and 284 U L^−1^ PGM (dark cyan line), and 104 U L^−1^ CDP and 284 U L^−1^ PGM (magenta line), respectively. Values shown are means of triplicate determinations

As shown in Fig. 5a, an in vitro synthetic enzymatic biosystem was designed to generate electricity from cellodextrins (average DP 4.4). G6P was produced from cellodextrin via the cascade reaction of CDP and PGM. Two subsequent cascade enzymes, G6PDH and 6-phosphogluconate dehydrogenase (6PGDH, EC 1.1.1.44), were used to generate two moles of NADH from one mole of G6P. NADH was subsequently re-oxidized by diaphorase (DI), producing two electrons per NADH. These electrons were shuttled to the anode via an electron mediator, 9,10-anthraquinone-2,7-disulfonic acid (AQDS). Thus, one molecule of glucose unit of cellodextrins produces four molecules of electrons in the anode. In the cathode, oxygen was reduced into water that was catalyzed by the carbon cloth with Pt. On the basis of the results in Fig. 4, type I enzyme complex with the highest enhancement value of the initial reaction rate, and type IV enzyme complex with the highest initial reaction rate, their corresponding free enzyme mixtures were evaluated for electrochemical performance in an anodic reaction system at 60 °C containing five enzymes (CDP, PGM, G6PDH, 6PGDH and DI), NAD^+^, inorganic phosphate, electron mediator AQDS and cellodextrin [43].

A 3-electrode system was used to determine the current density in the initial stage by measuring the amperometric *i*–*t* curve. A constant potential of 0.1 V was applied to determine the bioelectricity generated from cellodextrin (average DP 4.4). Because the bioelectricity generation (current density) refers to the reaction rate, and the maximum G6P production rate was maintained at the first 20 min (Fig. 4), thus within a time period of 20 min, the current density will reach its highest value. Therefore, the first 18 min was selected to determine the current density. Under the enzyme unit loading of 8.6 U L^−1^ CDP and 284 U L^−1^ PGM, the current density of the biosystem containing type I CDP–PGM complex was 0.096 mA cm^−2^ at 18 min, which was 3.35 times higher than that of the free enzyme mixture-based biosystem (Fig. 5b). Under the enzyme unit loading of 17.2 U L^−1^ CDP and 284 U L^−1^ PGM, the biosystem containing type IV CDP–PGM complex could give out 0.115 mA cm^−2^ current density at 18 min, about 2 times higher than that of the free enzyme mixture-based biosystem. Because the molar ratio of CDP and PGM was fixed at 1:1 and 2:1 in type I and type IV enzyme complex, respectively, the enzyme unit loading of PGM was much higher than that of CDP due to the high specific activity of PGM. Actually, we could just increase the CDP loading to enhance the initial current density in enzyme mixture. Under the PGM unit loading of 284 U L^−1^, when increasing the loading unit of CDP to 34.4 U L^−1^ and 103.2 U L^−1^ in free enzyme mixture-based biosystem, 0.089 and 0.11 mA cm^−2^ current density were obtained, respectively (Fig. 5b). However, initial current density of type I (8.6 U L^−1^ CDP and 284 U L^−1^ PGM) and type IV (17.2 U L^−1^ CDP and 284 U L^−1^ PGM) CDP–PGM complex-based biosystem was overmatched the performance of enzyme mixture-based biosystem containing 34.4 U L^−1^ CDP and 284 U L^−1^ PGM and enzyme mixture-based biosystem containing 103.2 U L^−1^ CDP and 284 U L^−1^ PGM, respectively. These results indicate that enzyme complexes can accelerate cascade biocatalysis and reduce enzyme loading to obtain the same catalytic efficiency in in vitro synthetic enzymatic biosystem for bioelectricity generation from cellodextrin.

Moreover, power density values using linear sweep voltammetry at a scan rate of 1 mV s^−1^ of the two biosystems containing CDP–PGM synthetic enzyme complex or free enzyme mixture were also determined. For type I synthetic enzyme complex containing 8.6 U L^−1^ CDP and 284 U L^−1^ PGM, the open circuit potential (OCP) was determined at 0.51 and 0.35 V, and a short connection current density was 1.03 and 0.74 mA cm^−2^ for the type I synthetic enzyme complex and its corresponding free enzyme mixture, respectively (Fig. 5c). The maximum power density of the biosystem mediated by the type I CDP–PGM complex was 0.084 mW cm^−2^, which is 2.14-fold higher than that of its corresponding free enzyme mixture-based biosystem (Fig. 5d). While, under the unit loading of 17.2 U L^−1^ CDP and 284 U L^−1^ PGM, the OCP was determined at 0.54 and 0.39 V, and a short connection current density was 1.12 and 0.86 mA cm^−2^ for the type IV synthetic enzyme complex and its corresponding free enzyme mixture, respectively (Fig. 5c). The maximum power density of the biosystems mediated by the type IV CDP–PGM complex and its corresponding free enzyme mixture was 0.1 and 0.057 mW cm^−2^, respectively. These results also showed that construction of CDP–PGM complex could increase the rate of bioelectricity generation by in vitro synthetic enzymatic biosystems using cellulosic biomass as starting material.

## Discussion

Cellulosic biomass has been proved to be a potential starting material in the production of biocommodities via in vitro synthetic enzymatic biosystem [19]. However, the initial reaction rate and conversion efficiency of this in vitro biosystem was limited by the high activation energy of whole enzyme cascade, positive Gibbs free energies convert cellodextrin to G1P (more than 2.4 kJ mol^−1^, Additional file 1: Table S1), low activity of CDP for phosphorolysis direction [37], and the poor pulling ability of downstream enzymes, especially in the hydrogen and bioelectricity production pathway. In this study, an example of accelerated cascade biocatalysis was demonstrated by constructing synthetic enzyme complexes in an in vitro synthetic enzymatic biosystem for bioelectricity generation. The demonstration of combining non-energetically favorable reaction with energetically favorable reaction via synthetic enzyme complex to accelerate bioelectricity generation from cellodextrin provides a feasible strategy on improving the efficiency of phosphorolysis of cellulosic biomass for biomanufacturing via in vitro synthetic enzymatic biosystems.

Four types of CDP–PGM complexes tested in this study all exhibited enhanced initial reaction rate and enhanced cellodextrin phosphorolysis (Fig. 4). Many exquisite designed multienzyme architectures, such as nanocaging [44], clustering [45] and protein scaffolds [46], exhibited promoted catalysis in artificial systems. However, the mechanism leading to accelerated product formation in spatial organized protein scaffolds is still widely debated. Frequently, the accepted hypothesis is protein scaffolds facilitate transfer of intermediates between enzyme cascade in artificial biosystems, which is called proximity effect that is supported by the spatial organization and compartmentalization [24, 47, 48]. On the contrary, Hess et al. [25] stated that the enhancement in initial reaction rate of synthetic enzyme complexes is caused by the alteration of enzyme characteristics in protein scaffold, but not the proximity of the enzymes. In this study, we test the apparent kinetic parameters of the enzyme complex and free enzyme mixture, which shows higher catalytic efficiency of the enzyme complex than that of the enzyme mixture (Table 1). However, no matter in enzyme complex or free enzyme mixture, the activity of PGM was much higher than CDP, so the apparent kinetic parameters of the enzyme complex and free enzyme mixture can be regarded as the kinetic parameters of CDP; thus the construction of enzyme complex increased the catalytic efficiency of CDP [25]. This result is corresponding to Hess’s point [25]. On the other side, the reaction rate of enzyme catalysis is the negative exponential function of activation energy [42], in this study, the activation energy value of enzyme complex was lower than that of free enzyme mixture, indicating that construction of enzyme complex may increase the reaction rate by lowering the activation energy. However, we could not exclude that the reason for enhanced activity of enzyme complex is the proximity effect of the enzymes. In the free enzyme mixture containing CDP and PGM, the interenzyme distance is much longer than the diffusion layer of enzymes [49]. The G1P molecules generated from the phosphorolytic cleavage of cellodextrin can be taken back by CDP for cellodextrin synthesis before reaching the PGM molecule to generate G6P (Fig. 1b), resulting in a low level of cellodextrin phosphorolysis. However, in the synthetic enzyme complexes containing CDP and PGM, the G1P molecules generated from the phosphorolytic cleavage of cellodextrin were quickly converted into G6P by proximal PGM without full equilibration with the bulk phase, leading to enhanced utilization rate of cellodextrin (Fig. 1c).

Four types of synthetic enzyme complexes were constructed with different spatial organizations of CDP and PGM (Fig. 2), showing different enhancement of initial rates compared with free enzyme counterparts (Fig. 4). Many factors affect the initial reaction rate of the synthetic complexes, such as interenzyme distance, enzyme orientation and multienzyme architecture [50]. The interenzyme distance of CDP and PGM of the type I complex is much shorter than in the type III complex, leading to a 50% increase in the initial reaction rate (17.3 vs. 11.4 μM min^−1^). For the construction of synthetic enzyme complexes, some experimental observations and molecular simulations of cascade enzyme reactions showed that the distance between cascade enzymes in the synthetic enzyme complex should neither be too long to decrease the enhancement of initial reaction rate nor too short to cause steric hindrance of the enzyme [47, 51–53]. The difference between type I and type II complexes is enzyme orientation, which results in different level of enhancement in initial reaction rate. This result may indicate that the active sites of CDP and PGM in the type I complex are more inward-facing than in the type II complex, allowing a high probability of the downstream reaction. The experimental descriptions of the effects of enzyme orientation in enzyme complexes have yet to be developed and represent an important area of future research for the investigation of the molecular mechanism of enzyme complexes [50]. Type IV synthetic complex contained 2 copies of CDP, and the initial reaction rate of the type IV CDP–PGM complex was much higher than that of the type I synthetic complex, but the enhancement of initial reaction rate of type IV synthetic enzyme complex was much lower than type I synthetic enzyme complex (Fig. 4c). Two copies of CDP could increase the local concentration of G1P, and high substrate concentration may lead to a low level of enhancement in initial reaction rate [38]. The optimization of the spatial organization of the enzyme complex is necessary to overcome barriers caused by inefficient enzymes. Although constructing the synthetic enzyme complex through the interaction between cohesins and dockerins is more robust, more engineerable and easier than nucleic acid-based construction [54], it would be highly beneficial to develop a computational method for the in silico prediction of effects of different spatial organizations prior to time-consuming construction and testing in the lab [24, 50]. Type I CDP–PGM complex, which showed the highest enhancement value of the initial reaction rate, was applied to an in vitro synthetic enzymatic biosystem for bioelectricity generation from cellodextrin, exhibiting an initial reaction rate 3.35 times higher than the non-complexed enzyme mixture (Fig. 5b). The enhancement of initial reaction rate of the type I CDP–PGM complex in the electricity generation pathway is higher than that in the G6P generation pathway (3.35 vs. 2.4) at the same enzyme concentration, which was possibly due to the further consumption of G6P by downstream enzymes for electricity generation.

The polarization curve represents the basic kinetics of the electrochemical system, which should be controlled by the rate-limiting step including enzymatic catalysis, electron transfer, mass transfer and so on. In Fig. 5c, all four curves show an obvious activation loss region at low current density region, revealing that the system is mainly controlled by the electrochemical process, more specifically the oxidation of G6P. Besides, a nice Ohmic loss region was shown in the region of middle current density, in which the voltage altered linearly with the current density followed by Ohm’s law. However, only a slight concentration loss can be observed at high current density region, suggesting that this bioelectricity production biosystem has relatively good mass transfer. Besides, both the power density and current density were increased with the increase of the CDP loading to 17.2 U L^−1^ (Fig. 5c, d). At faster transfer of G1P from CDP to PGM, the higher current density can be observed as shown in the case for the enzyme complex. Therefore, the amount of CDP and thus the availability of G6P converted from G1P and cellodextrin should be the key rate-limiting step in this bioelectrochemical system.

In addition, compared with type I enzyme complex, increase the copy number of CDP in our enzyme complex will continue to improve the performance of this cellodextrin-powered biosystem, just like type IV enzyme complex in the Fig. 5. But inevitably, when the number of cohesin copies is more than five, the scaffoldin protein is too large to be stable and difficult to heterologous express in *Escherichia coli*. Of course, we can construct multi-level scaffoldin in the future work to increase the copy number of CDP in enzyme complex like the assemble structure of natural cellulosomes [55]. However, as the number of CDP copies increases in the synthetic enzyme complex, the enhancement value of power output of bioelectricity generation by in vitro synthetic enzymatic biosystems will decrease and the effect of reduced enzyme loading by construction of enzyme complex will become diminished (Fig. 5). Thus, an appropriate number of CDP copies need to be further investigated in the enzyme complex to balance the economic benefits between power output values and enzyme loading amounts in bioelectricity generation from cellodextrin by in vitro synthetic enzymatic biosystems. Besides, the integration of more enzymes such as G6PDH and 6PGDH into the synthetic enzyme complexes may further increase the reaction rate of bioelectricity generation from cellodextrin.

## Conclusions

In this study, we first revealed an in vitro synthetic enzymatic biosystems for the bioelectricity production from cellulosic biomass. In addition, our results successfully demonstrate that constructing CDP–PGM synthetic enzyme complex is a feasible approach to overcome thermodynamic and activation barriers in cellodextrin phosphorolysis and enhance the power output of cellodextrin-powered in vitro synthetic enzymatic biosystem. This CDP–PGM complex can also be used to increase the efficiency of other in vitro synthetic biosystems using cellodextrin as starting material to produce hydrogen [14] and other value-added chemicals such as inositol [19]. For the aim of applying of synthetic enzyme complex for cellulose utilization through phosphorolysis, several efforts are urgently-needed including the clarify of deep mechanism of synthetic enzyme complex, finding new CDPs with high phosphorolysis activity, as well as rational design of efficiency enzyme complex before testing in lab.

## Methods

### Chemicals, strains and medium

All chemicals were reagent grade and purchased from Sigma-Aldrich (St. Louis, MO) or Sinopharm (Shanghai, China) unless otherwise noted. Microcrystalline cellulose Avicel PH-105 was purchased from FMC (Philadelphia, PA, US). Regenerated amorphous cellulose (RAC) was prepared from Avicel PH-105 through cellulose dissolution and precipitation [56]. Cellodextrin was prepared by mixed-acid hydrolysis from Avicel PH-105 as previously reported [5]; the average degree of polymerization (DP) of cellodextrin used in this study is 4.4 [19]. The PCR enzyme used was Phusion high-fidelity DNA polymerase from New England Biolabs (Ipswich, MA, US). Oligonucleotide primers and DNA sequencing were performed at Genewiz (Suzhou, China). *E. coli* DH5α was used for plasmid maintenance, and *E. coli* BL21 (DE3) was used for gene expression.

### Construction of plasmids

Primers in Table 2 were used to amplify DNA fragments on corresponding template to prepare plasmids. All the plasmids used in this study are listed in Table 3.

**Table 2:** Primers used in this study

Primer	Sequence (5′–3′)	Template
VF-CDP-CtDoc	acatatagtgactcttaagtttaaagttgattggaacaaggcaactgctt	pET20b-tim-ctdoc
VR-CDP-CtDoc	aagagcgacaatgaaatcattagtatacatatagaggaagaatttcaatt	
IF-CDP-CtDoc	ttaactttaagaaggagatatacatatgattactaaagtaacagcgagaa	pET21c-ctcdp
IR-CDP-CtDoc	ttcgtcaacggaacaaggttagttgaaatttgaattctcagtgatataca	
VF-CDP-RfDoc	acatatagtgactcttaagtttaaacccggcacaaagctcgttcctacat	pET20b-fbp-rfdoc
VR-CDP-RfDoc	The same with VR-CDP-CtDoc	
IF-CDP-RfDoc	The same with IF-CDP-CtDoc	pET21c-ctcdp
IR-CDP-RfDoc	tacatccttgctcgaaacacggcccaaatttgaattctcagtgatataca	
VF-PGM-CcsDoc	actgctggaagaagcactgaaaggtggtaaggtattaccaggaatccaag	pET20b-ald-ccsdoc
VR-PGM-CcsDoc	gcttccatggtttgtcaaacgggtatacatatagaggaagaatttcaatt	
IF-PGM-CcsDoc	ttaactttaagaaggagatatacatatgggcaaactgtttggtaccttcg	pET20b-tkpgm
IR-PGM-CcsDoc	gaacctaaggaccattatggaatggtggaaagtcacgaagaaggtcgtca	
VF-PGM-RfDoc	actgctggaagaagcactgaaaggtcccggcacaaagctcgttcctacat	pET20b-fbp-rfdoc
VR-PGM-RfDoc	The same with VR-PGM-CcsDoc	
IF-PGM-RfDoc	The same with IF-PGM-CcsDoc	pET20b-tkpgm
IR-PGM-RfDoc	tacatccttgctcgaaacacggccctggaaagtcacgaagaaggtcgtca

**Table 3:** Plasmids used in this study

Plasmids	Characteristics	Refs.
pET20b-cbm3-scaf3	Amp^R^, mini-scaffoldin expression cassette containing a CBM3 module from *C. thermocellum* CipA and three different cohesins from *C. thermocellum*, *C. cellulovorans* and *R. flavefaciens*	[40]
pET21c-ctcdp	Amp^R^, ctcdp expression cassette containing CDP, which was purified based on the C-terminal 6× His tag	[37]
pET20b-tkpgm	Amp^R^, tkpgm expression cassette containing PGM, which was purified based on the C-terminal 6× His tag	[10]
pET20b-ctcdp-ctdoc	Amp^R^, ctcdp-ctdoc expression cassette containing CDP from *T. thermophiles* and the dockerin module from *C. thermocellum*	This work
pET20b-ctcdp-rfdoc	Amp^R^, ctcdp-rfdoc expression cassette containing CDP module from *T. maritime* and the dockerin module from *R. flavefaciens*	This work
pET20b-tkpgm-ccsdoc	Amp^R^, tkpgm-ccsdoc expression cassette containing PGM module from *T. maritime* and the dockerin module from *C. cellulovorans*	This work
pET20b-tkpgm-rfdoc	Amp^R^, tkpgm-rfdoc expression cassette containing PGM module from *T. maritime* and the dockerin module from *R. flavefaciens*	This work

Plasmid pET20b-cbm3-scaf3, which has an expression cassette containing one CBM3 module and three cohesin modules from *C. thermocellum* ATCC 27405, *C. cellulovorans* ATCC 35296 and *R. flavefaciens*, respectively, was obtained from our previous work [40]. Plasmid pET21c-ctcdp had a ctcdp gene (Cthe2989) encoding cellodextrin phosphorylase from *C. thermocellum* and a C-terminal 6× His tag expression gene [37]. Plasmid pET20b-tkpgm had a tkpgm gene (Tk1108) encoding phosphoglucomutase from *T. kodakaraensis* and a C-terminal 6× His tag expression gene [10].

Plasmid pET20b-ctcdp-ctdoc contained an expression cassette of the ctcdp gene and a dockerin module from *C. thermocellum* CelS (CtDoc, 673–741 amino acids, GenBank Accession number: L06942). The ctcdp gene was amplified from the plasmid pET21c-ctcdp using a primer pair of IF-CDP-CtDoc and IR-CDP-CtDoc; the pET20b-ctdoc gene backbone was amplified from plasmid pET20b-tim-ctdoc [40] with a primer pair of VF-CDP-CtDoc and VR-CDP-CtDoc. Plasmid pET20b-ctcdp-ctdoc based on these two DNA fragments was obtained by Simple Cloning [57].

Plasmid pET20b-ctcdp-rfdoc contained an expression cassette of the ctcdp gene and a dockerin module from *R. flavefaciens* ScaA (RfDoc, 787–879 amino acids, GenBank Accession number: CAC34384.3). The ctcdp gene was amplified from the plasmid pET21c-ctcdp using a primer pair of IF-CDP-RfDoc and IR-CDP-RfDoc; the pET20b-rfdoc gene backbone was amplified from plasmid pET20b-fbp-rfdoc [40] with a primer pair of VF-CDP-RfDoc and VR-CDP-RfDoc. Plasmid pET20b-ctcdp-rfdoc was obtained by Simple Cloning.

Plasmid pET20b-tkpgm-ccsdoc contained an expression cassette of the tkpgm gene and a dockerin module from *C. cellulovorans* EngE (CcsDoc, 943–1030 amino acids, GenBank Accession number: AAD39739.1). The tkpgm gene was amplified from the plasmid pET20b-tkpgm using a primer pair of IF-PGM-CcsDoc and IR-PGM-CcsDoc; pET20b-ccsdoc gene backbone was amplified from plasmid pET20b-ald-ccsdoc [40] with a primer pair of VF-PGM-CcsDoc and VR-PGM-CcsDoc. Plasmid pET20b-tkpgm-ccsdoc was obtained by Simple Cloning.

The pET20b-tkpgm-rfdoc plasmid contained an expression cassette of tkpgm gene and the RfDoc module from *R. flavefaciens* ScaA. The tkpgm gene was amplified from the plasmid pET20b-tkpgm using a primer pair of IF-PGM-RfDoc and IR-PGM-RfDoc; pET20b-rfdoc gene backbone was amplified from the plasmid pET20b-fbp-rfdoc [40] with a primer pair of VF-PGM-RfDoc and VR-PGM-RfDoc. The pET20b-tkpgm-rfdoc plasmid was obtained by Simple Cloning.

### Overexpression and purification of recombinant proteins

The strains *E. coli* BL21 Star (DE3) containing the protein expression plasmids were cultivated in the Luria–Bertani (LB) medium supplemented with 100 mg L^−1^ ampicillin at 37 °C. When OD_600nm_ reached about 0.75, 100 µM isopropyl-beta-d-thiogalactopyranoside (IPTG, a final concentration) was added and the temperature was decreased to 16 °C for ~ 16 h. After centrifugation, the cell pellets were resuspended in 50 mM HEPES buffer (4-(2-hydroxyethyl)-1-piperazineethanesulfonic acid) (pH 7.5) containing 1 mM CaCl_2_ and 50 mM NaCl. The cells were lysed by ultrasonication. After centrifugation at 10,000*g* for 15 min, 10 µL of the supernatant was loaded into 12% SDS-PAGE to check the expression level of the proteins. His-tagged protein was purified by Ni–NTA resin. Protein concentrations were determined using the Bradford method with bovine serum albumin as the standard [58].

### One-step purification and immobilization for synthetic enzyme complex

Four kinds of cell lysate supernatant, CDP-CtDoc (1 mL), CDP-RfDoc (1.2 mL), PGM-CcsDoc (1.2 mL), PGM-RfDoc (1 mL), were selectively mixed with 0.5 mL of the cell lysate supernatant of CBM-Scaf3. 2 mg RAC was then added to the mixture to adsorb the CBM3-containing enzyme complex at room temperature for 5 min. After centrifugation at 5000*g* for 10 min, the RAC pellet was washed in 100 mM HEPES (pH 7.5) containing 50 mM NaCl and 1 mM CaCl_2_ three times and then was checked by 12% SDS-PAGE for the binding profiles. The RAC pull-down protein mass based on the Bradford method was calibrated by their absorbance (280 nm) in 6 M guanidine hydrochloride [40].

### Enzymatic activity assays

The enzymatic activity of CDP was measured in 100 mM HEPES buffer (pH 7.5) containing 5 mM MgCl_2_, 5 mM dithiothreitol (DTT), 10 mM KH_2_PO_4_ and 5 g L^−1^ cellodextrin at 60 °C. The reaction was stopped by boiling for 5 min. The product G1P was measured using a coupled spectrophotometric assay with sufficient recombination PGM and G6PDH in the presence of NAD^+^, and the generation of NADH was measured at 340 nm. One unit of enzyme activity was defined as the amount of enzyme that released 1 μmol of G1P per minute. Unless otherwise stated, each measurement was conducted in triplicate.

The enzymatic activity of PGM was measured in 100 mM HEPES buffer (pH 7.5) containing 5 mM MgCl_2_ and 5 mM G1P at 60 °C. The reaction was stopped by boiling for 5 min. The product G6P was determined by using a glucose hexokinase/glucose-6-phosphate dehydrogenase (HK⁄G6PDH) assay kit (Sigma-Aldrich). One unit of enzyme activity was defined as the amount of enzyme that released 1 μmol of G6P per minute.

The cascade enzyme activity was measured in 100 mM HEPES buffer (pH 7.5) containing 5 mM MgCl_2_, 5 mM dithiothreitol (DTT), 10 mM KH_2_PO_4_ and 5 g L^−1^ cellodextrin at 60 °C. Appropriate amounts of RAC immobilized bifunctional synthetic enzyme complex and free enzyme mixture were tested to determine the enzymatic activity to calculate the initial reaction rate. Samples without enzyme or substrate were used as negative controls. Samples were withdrawn at different time points, terminated by adding perchloric acid and neutralized with KOH [40]. The production of G6P was measured using a coupled spectrophotometric assay with sufficient recombination G6PDH in the presence of NAD^+^. The enhancement of initial reaction rate was defined as the ratio of the initial reaction rate of the synthetic enzyme complex to that of the free enzyme mixture at the same enzyme activity loading. Michaelis–Menten kinetic parameters of the synthetic enzyme complex and free enzyme mixture against cellodextrin and cellopentaose were determined, respectively. The reaction was performed at 60 °C and G6P was measured as previously described. The apparent kinetic parameters were estimated using the Michaelis–Menten equation with GraphPad Prism 5.01 software (San Diego, CA) by employing nonlinear regression. Activation energy of enzyme mixture and enzyme complex were determined based on the slope of Arrhenius plot, which was depicted as ln(*k*_cat_) versus 1/*T* (K).

### Electrochemical measurement

All electrochemical tests were performed using a CHI660E electrochemical workstation (Shanghai Chenhua Instrument, China) interfaced with a computer. All measurements were performed at 60 °C in a 5-mL anolyte with 100 mM HEPES buffer (pH 7.5) containing 5 mM MgCl_2_, 5 mM DTT, 20 mM KH_2_PO_4_, 10 mM NAD^+^, 10 mM AQDS, 5 g L^−1^ cellodextrin (average DP 4.4), appropriate amounts of RAC immobilized bifunctional enzyme complex or enzyme mixture, 0.015 mg mL^−1^ of thermostable mutant *Zymomonas mobilis* G6PDH [59], 0.015 mg mL^−1^ of *Moorella thermoacetica* 6PGDH [60] and 0.015 mg mL^−1^ of *Geobacillus stearothermophilus* DI [9]. The reaction systems in the absence of cellodextrin or enzymes were adopted as two control systems.

To determine the current density in the initial stage, the amperometric *i*–*t* curve was measured at a constant applied potential of 0.1 V using a 3-electrode system with a glassy carbon working electrode, an Ag/AgCl reference electrode and a Pt wire counter electrode. The RAC immobilized bifunctional enzyme complex or non-immobilized enzyme mixture was tested in the anolyte under 60 °C for 18 min, respectively.

Linear sweep voltammetry (LSV) has been widely used in the field of characterization of bioelectricity production [61–63]. In this study, LSV was performed at a scan rate of 1 mV s^−1^ in a bioelectricity generating configuration to measure the power density of the in vitro synthetic enzymatic biosystem catalyzed by the RAC immobilized bifunctional synthetic enzyme complex and non-immobilized enzyme mixture. A “I-cell” cuvette biosystem was set up as described previously, which was assembled by two stacked glass tubes [43, 64]. A 1-cm^2^ carbon felt anode was dipped in the enzyme-containing anolyte that served in the upper glass tube. The carbon cloth was coated with 0.5 mg cm^−2^ Pt as an air-breathing cathode for oxygen reduction, which was sealed by O-rings in the middle of two glass tubes. Nafion 212 was used as the proton exchange membrane to separate the two electrodes. The performance of the in vitro synthetic enzymatic biosystem with the immobilized synthetic enzyme complex and non-immobilized enzyme mixture was evaluated after 4 h reaction at 60 °C, respectively.

## Supplementary information

**Additional file 1: Table S1.** Reaction Gibbs free energy of cellodextrin phosphorolysis. **Table S2.** Apparent kinetic parameters for the type I synthetic enzyme complex and enzyme mixture under different temperature. **Figure S1.** Profiles of enzyme activity in RAC immobilized enzyme complex. **Figure S2.** Characterization of type I enzyme complex in high enzyme loading. **Figure S3.** Effect of TmPase on the cascade reaction of CDP and PGM.

## Data Availability

All data generated or analyzed during this study are included in this published article and its additional files.

## Abbreviations

G1P: glucose 1-phosphate
G6P: glucose 6-phosphate
CDP: cellodextrin phosphorylase
PGM: phosphoglucomutase
G6PDH: glucose-6-phosphate dehydrogenase
6PGDH: 6-phosphogluconate dehydrogenase
DI: diaphorase
HK/G6PDH: hexokinase/glucose-6-phosphate dehydrogenase
LSV: linear sweep voltammetry
GH: glycoside hydrolase
RAC: regenerated amorphous cellulose
DP: degree of polymerization
LB: Luria–Bertani
IPTG: isopropyl-beta-d-thiogalactopyranoside
DTT: dithiothreitol
HEPES: 4-(2-hydroxyethyl)-1-piperazineethanesulfonic acid
AQDS: 9,10-anthraquinone-2,7-disulfonic acid
6PG: 6-phospho-d-glucono-1,5-lactone
I1P: inositol 1-phosphate
CBM3: family 3 cellulose-binding module
*k*_cat_/*K*_m_: catalytic efficiency
OCP: open circuit potential
