# Supplementary material for: Acceleration of cellodextrin phosphorolysis for bioelectricity generation from cellulosic biomass by integrating a synthetic two-enzyme complex into an in vitro synthetic enzymatic biosystem
Source: Biotechnol Biofuels. 2019 Nov 12;12:267. doi: 10.1186/s13068-019-1607-4 (PMC6849236; doi:10.1186/s13068-019-1607-4)
Supplement: Supplementary file 1 — Additional file 1: Table S1. Reaction Gibbs free energy of cellodextrin phosphorolysis. Table S2. Apparent kinetic parameters for the type I synthetic enzyme complex and enzyme mixture under different temperature. Figure S1. Profiles of enzyme activity in RAC immobilized enzyme complex. Figure S2. Characterization of type I enzyme complex in high enzyme loading. Figure S3. Effect of TmPase on the cascade reaction of CDP and PGM. [file 13068_2019_1607_MOESM1_ESM.docx]

**Additional file 1**

**Acceleration of cellodextrin phosphorolysis for bioelectricity generation from cellulosic biomass by integrating a synthetic two-enzyme complex into an in vitro synthetic enzymatic biosystem**

Dongdong Meng^1^, Ranran Wu^1^, Juan Wang^1^, Zhiguang Zhu^1,2*^, Chun You^1,2*^

^1^ Tianjin Institute of Industrial Biotechnology, Chinese Academy of Sciences, 32 West 7th Avenue, Tianjin Airport Economic Area, Tianjin 300308, People’s Republic of China

^2^ University of Chinese Academy of Sciences, Beijing, China

*Corresponding author: Chun You, Tianjin Institute of Industrial Biotechnology, Chinese Academy of Sciences, Tianjin 300308, China. Tel.: 86-22-24828795, E-mail: you_c@tib.cas.cn; Zhiguang Zhu, Tianjin Institute of Industrial Biotechnology, Chinese Academy of Sciences, Tianjin 300308, China. Tel.: 86-22-24828795, E-mail: [zhu_zg@tib.cas.cn](mailto:zhu_zg@tib.cas.cn)

**Table S1** Reaction Gibbs free energy of cellodextrin phosphorolysis at pH 7.0 and 0.1 M ionic strength, the data is obtained from <http://equilibrator.weizmann.ac.il/>.

| **Reaction** | **Estimated** [**Δ_r_G'°**](http://equilibrator.weizmann.ac.il/static/classic_rxns/faq.html#drg) **(KJ mol^-1^)** |
| --- | --- |
| [Cellohexao](http://equilibrator.weizmann.ac.il/compound?compoundId=C06217)se + [Phosphate](http://equilibrator.weizmann.ac.il/compound?compoundId=C00009) ⇌ [Cellopentaose](http://equilibrator.weizmann.ac.il/compound?compoundId=C06218) + [Glucose 1-phosphate](http://equilibrator.weizmann.ac.il/compound?compoundId=C00103) | **3.2** ± 3.6 |
| [Cellopentaose](http://equilibrator.weizmann.ac.il/compound?compoundId=C06218) + [Phosphate](http://equilibrator.weizmann.ac.il/compound?compoundId=C00009) ⇌ Cellotetraose + [Glucose 1-phosphate](http://equilibrator.weizmann.ac.il/compound?compoundId=C00103) | **3.2** ± 3.6 |
| Cellotetraose + [Phosphate](http://equilibrator.weizmann.ac.il/compound?compoundId=C00009) ⇌ Cellotriose + [Glucose 1-phosphate](http://equilibrator.weizmann.ac.il/compound?compoundId=C00103) | **2.4** ± 13.7 |
| Cellotriose + Phosphate ⇌ Cellobiose + [Glucose 1-phosphate](http://equilibrator.weizmann.ac.il/compound?compoundId=C00103) | **2.8** ± 5.8 |

**Table S2 Apparent kinetic parameters for the type I synthetic enzyme complex and enzyme mixture under different temperature against cellodextrins.**

|  | | *K*_m_  (g L^-1^) | *k*_cat_  (s^-1^) | *k*_cat_/*K*_m_  (s^-1^ g^-1^ L) | Activation energy (kJ mol^-1^) |
| --- | --- | --- | --- | --- | --- |
| Type I CDP−PGM complex | 45 ^o^C | 3.4 ± 0.3 | 2.3 ± 0.1 | 0.68 | 32.8 ± 0.7 |
|  | 50 ^o^C | 2.6 ± 0.2 | 2.8 ± 0.1 | 1.05 |  |
|  | 55 ^o^C | 2.7 ± 0.2 | 3.7 ± 0.1 | 1.37 |  |
|  | 60 ^o^C | 2.1 ± 0.7 | 3.9 ± 0.4 | 1.9 |  |
| Enzyme mixture | 45 ^o^C | 3.6 ± 0.3 | 0.65 ± 0.03 | 0.18 | 45.8 ± 1.8 |
|  | 50 ^o^C | 2.9 ± 0.4 | 0.99 ± 0.06 | 0.33 |  |
|  | 55 ^o^C | 3.8 ± 0.5 | 1.22 ± 0.1 | 0.32 |  |
|  | 60 ^o^C | 2.8 ± 0.3 | 1.5 ± 0.1 | 0.55 |  |

**
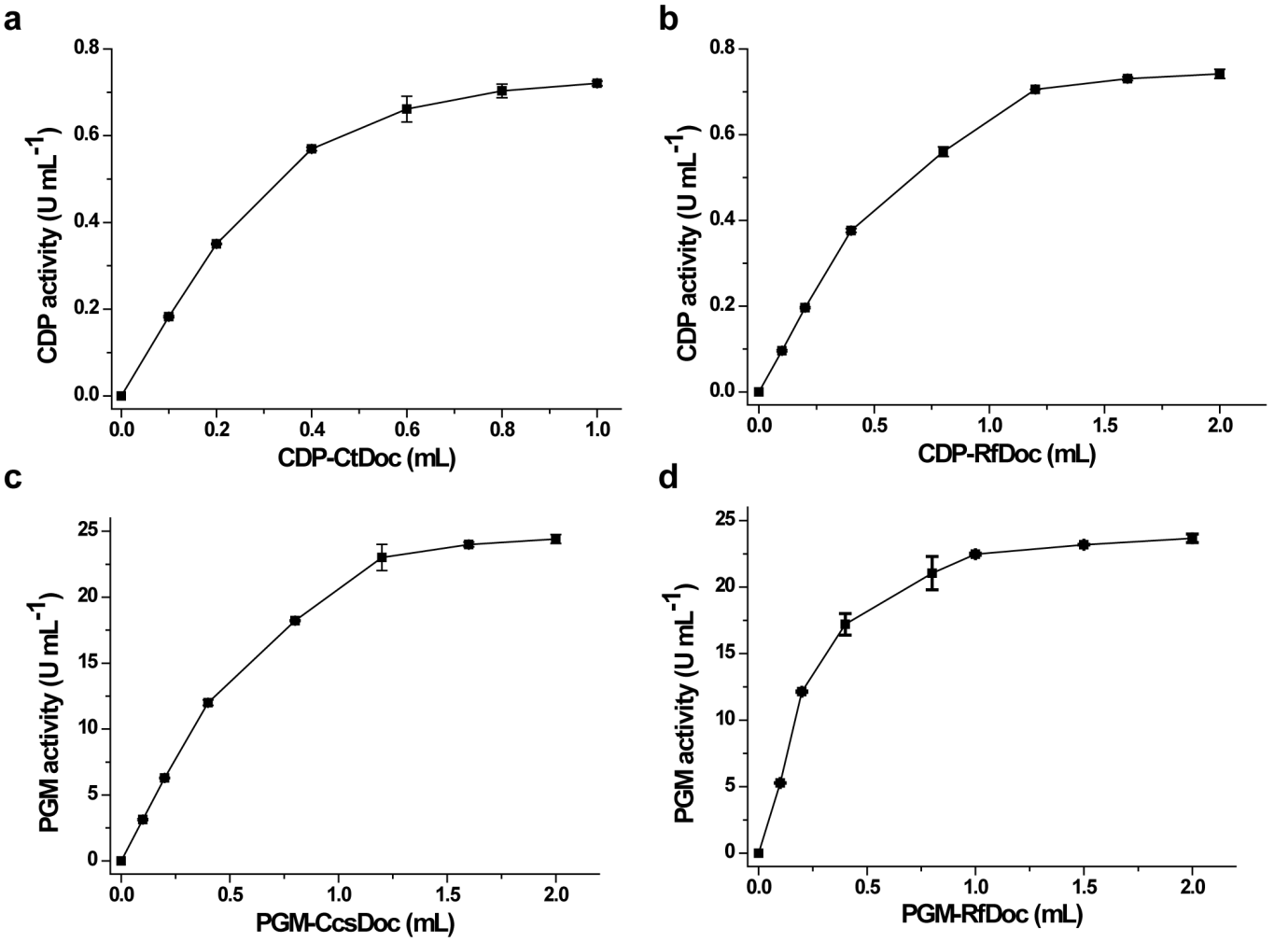
**

**Figure S1**. Profiles of enzyme activity in RAC immobilized CBM3-Scaf3/CDP-CtDoc (A), CBM3-Scaf3/CDP-RfDoc (B), CBM3-Scaf3/PGM-CcsDoc (C), and CBM3-Scaf3/PGM-RfDoc (D) against the amount of cell lysate supernatant of dockerin-tagged enzyme that used for enzyme complex construction. Values shown are means of triplicate determinations.


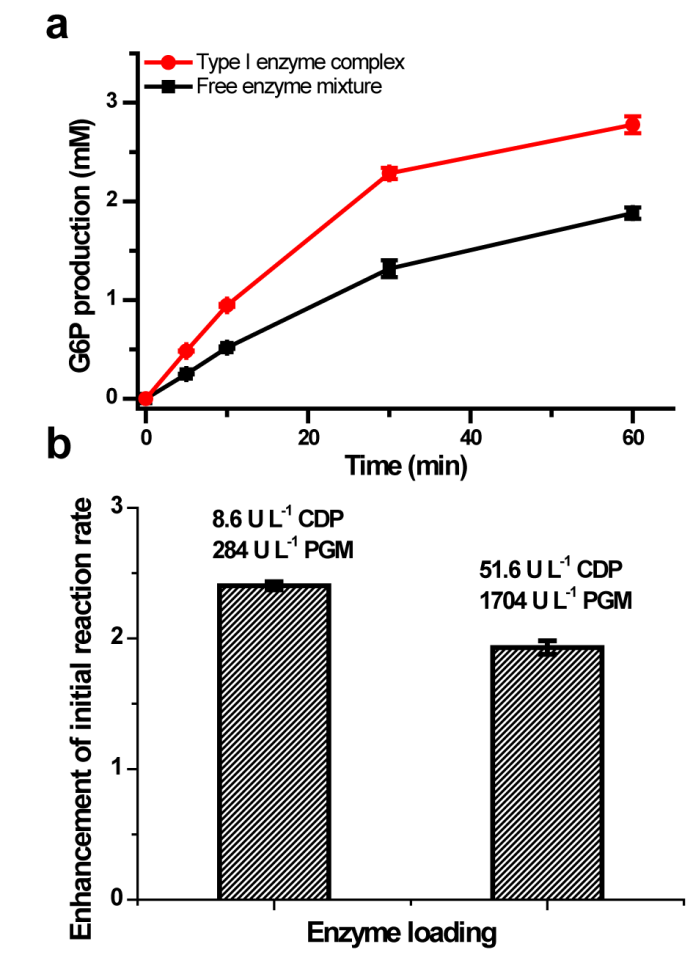


**Figure S2** (A) Profiles of G6P production catalyzed by type I CDP-PGM synthetic enzyme complex (circle) and free enzyme mixture (square) with the same enzyme unit loading of 51.6 U L^-1^ CDP and 1704 U L^-1^ PGM. (B) Values of enhancement in initial reaction rate of type I enzyme complex under different enzyme loading concentration. Values shown are means of triplicate determinations.

**
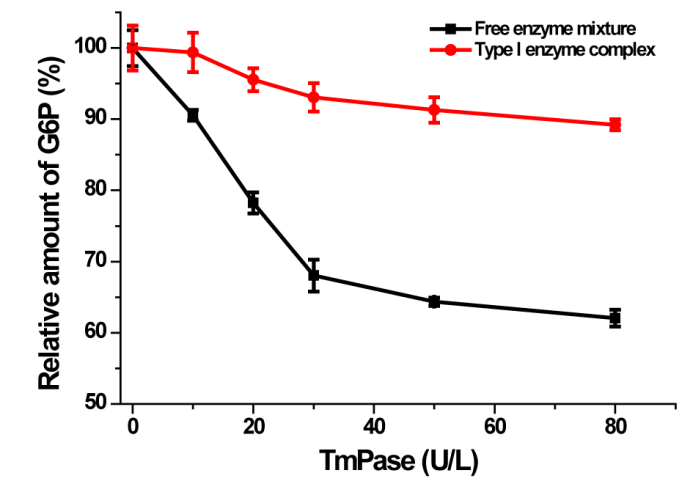
**

**Figure S3** Effect of *Thermotoga maritima* phosphatase (TmPase) on the cascade reaction of CDP and PGM. The reaction was performed in 100 mM HEPES buffer (pH 7.5) containing 5 mM MgCl_2_, 5 mM dithiothreitol (DTT), 10 mM KH_2_PO_4_, and 5 g L^-1^ cellodextrin at 60 °C with the enzyme unit loading of 8.6 U L^-1^ CDP and 284 U L^-1^ PGM in the enzyme mixture or type I enzyme complex biosystem. Different units of TmPase (0 U L^-1^ to 80 U L^-1^) were added into the reactant and the amounts of G6P were determined after the reaction proceed at 10 min. The amount of G6P in the absence of TmPase was considered 100%. Values shown are means of triplicate determinations.
